# Engineering substrate promiscuity in halophilic alcohol dehydrogenase (*Hv*ADH2) by *in silico* design

**DOI:** 10.1371/journal.pone.0187482

**Published:** 2017-11-30

**Authors:** Jennifer Cassidy, Larah Bruen, Elena Rosini, Gianluca Molla, Loredano Pollegioni, Francesca Paradisi

**Affiliations:** 1 Synthesis and Solid State Pharmaceutical Centre (SSPC), School of Chemistry, University College Dublin, Belfield, Dublin, Ireland; 2 Dipartimento di Biotecnologie e Scienze della Vita, Università degli Studi dell'Insubria, Varese, Italy; 3 The Protein Factory, Politecnico di Milano, Università degli Studi dell'Insubria, Milano, Italy; 4 School of Chemistry, University Park, University of Nottingham, Nottingham, United Kingdom; Università degli Studi di Milano, ITALY

## Abstract

An alcohol dehydrogenase from the halophilic archaeon *Haloferax volcanii* (*Hv*ADH2) has been engineered by rational design to broaden its substrate scope towards the conversion of a range of aromatic substrates, including flurbiprofenol, that is an intermediate of the non-steroidal anti-inflammatory drug, flurbiprofen. Wild-type *Hv*ADH2 showed minimal activity with flurbiprofenol (11.1 mU/mg). A homology model of *Hv*ADH2 was built and docking experiments with this substrate revealed that the biphenyl rings of flurbiprofenol formed strong interactions with residues F85 and F108, preventing its optimal binding in the active site. Mutations at position 85 however did not increase activity. Site directed mutagenesis at position F108 allowed the identification of three variants showing a significant (up to 2.3-fold) enhancement of activity towards flurbiprofenol, when compared to wild-type *Hv*ADH2. Interestingly, F108G variant did not show the classic inhibition in the presence of (*R*)-enantiomer when tested with *rac*-1-phenylethanol, underling its potential in racemic resolution of secondary alcohols.

## Introduction

Enzymes are appealing as a ‘green’ adjunct to chemical synthesis of pharmaceutical building blocks because of their broad specificity, enantioselectivity and ability to work under process conditions [1]. Found in all three domains of life, alcohol dehydrogenases (ADHs) are members of the oxidoreductase family, which catalyze the interconversion of primary and/or secondary alcohols into aldehydes and ketones, respectively [2]. A recent review details the staggering applications of ADHs in the production of pharmaceutical building blocks [3]. Codexis described the evolution of *Lactobacillus kefir* ADH towards the enantiopure intermediate of the anti-depressant, (*S*)-duloxetine, with yields as high as 150 g/L [4].

Many additional examples of enzyme engineering applied to ADHs, which have led to variants suitable for industrial applications, have been reported in the literature [5–7]. Sequence alignments of protein families identify potential ‘hot-spots’ for mutagenesis as non-conserved positions; they are then further probed by homology modelling and *in silico* docking [8]. A site-directed mutagenesis strategy has applied for redesigning substrate specificity in glutamate dehydrogenase from *Halobacterium salinarum* [9]. The substitutions K89L, A163L and S367A converted this enzyme into a dehydrogenase accepting L-methionine, L-norleucine and L-norvaline as substrates. Biocatalytic strategies employing ADHs have already been reported to produce 2-arylpropionic acids and the corresponding derivatives [10–13]. Hyperthermophilic *Ss*ADH-10 from *Sulfolobus solfataricus* was applied to the enzymatic reduction and racemization of 2-arylpropionaldehydes [14].

In a previous study, *Hv*ADH2 from *Haloferax volcanii* (wild-type, WT) showed an unusually broad substrate specificity, with good activity with medium-chain alcohols, modest activity with secondary alcohols and also significant activity with benzyl alcohol [15–17]. Later studies into the solvent tolerance and immobilization of *Hv*ADH2 prompted a deeper investigation of the substrate scope of *Hv*ADH2 [18–19]. *Hv*ADH2 showed some activity with flurbiprofenol in a low concentration salt buffer to facilitate solubility.

## Results and discussion

### *Hv*ADH2 homology model and docking analysis

Previous characterization of *Hv*ADH2 showed that the enzyme has a broad substrate scope because it can accept medium-chain alcohols, has modest activity with secondary alcohols and retained 50% activity with the aromatic substrate, benzyl alcohol [17]. The potential of *Hv*ADH2 to reduce prochiral aromatic ketones was also investigated and it was found that 2-phenylpropionaldehyde was readily accepted (S7 Fig). The model of the 3D structure of *Hv*ADH2 was obtained by the SWISS-MODEL web-based server [20, 21] using the formaldehyde dismutase from *Pseudomonas putida* (PDB: 2dph) as the template (27% sequence identity with *Hv*ADH2) [22]. The quality of the *Hv*ADH2 homology model was assessed using the software ERRAT [23]: 30.1% of the protein structure model could be rejected at a 95% confidence level (as compared to a threshold of 5% rejection for a high quality model), but these (less accurate) regions are located on the protein surface and do not affect the overall conformation and substrate binding at the active site (S1 Fig). The quality of the model was assessed by several bioinformatic tools that confirmed it as a reliable model (see S2 Fig).

The NAD^+^ cofactor and the conserved catalytic Zn^2+^ ion were modelled into the *Hv*ADH2 model based on their position and conformation observed in the structure of the formaldehyde dehydrogenase from *Pseudomonas putida* (PDB: 1kol, 26% sequence identity with *Hv*ADH2) [24]. *Hv*ADH2 possesses the typical Rossmann binding motif found in NAD-binding enzymes.

**Fig 1:**
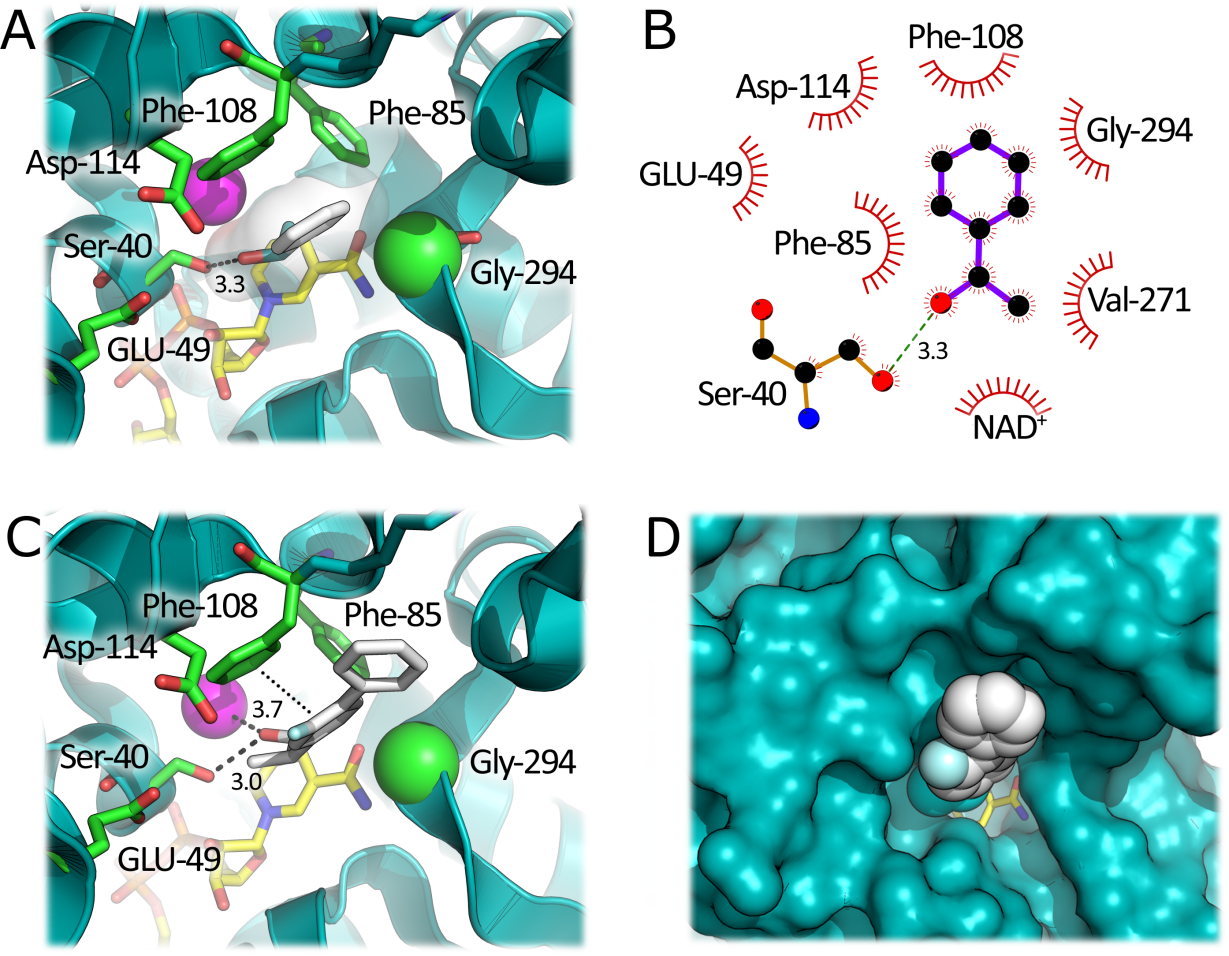
Three-dimensional model of *Hv*ADH2 active site in complex with different docked ligands. A) Complex with (*S*)-1-PheOH. B) LigPlot analysis of the interactions between docked (*S*)-1-PheOH and HvADH2 model. The substrate is in purple. Hydrophobic contacts are shown as dark red arches. C) Complex with (*S*)-flurbiprofenol. D) Complex with (S)-flurbiprofenol, surface representation. The ligands are represented as white sticks or spheres. The NAD^+^ cofactor is in yellow and Zn^2+^ is represented as a pink sphere. Important residues are shown in green; the αC of G294 is shown as a sphere. The π-π staking interactions are shown by dotted lines and H-bonds by dashed lines.

Docking studies with the secondary aromatic alcohol, (*S*)*-*1-phenylethanol, ((*S*)-1-PheOH) allowed the identification of the residues involved in the substrate binding at the active site of *Hv*ADH2. Two phenylalanine residues in position 85 and 108 (F85 and F108), at the top of the active site, were identified as being important for substrate binding. These residues, together with G294, form a hydrophobic pocket for the bulky aromatic ring of the substrate: in particular, F108 participates in π-π stacking interactions with the aromatic ring of (*S*)-1-PheOH. The polar hydroxyl group of the substrate is located in a polar region of the active site lined by E49, D144 and S40 which forms a H-bond with oxygen of (*S*)-1-PheOH (Fig 1). *Hv*ADH2 showed a specific activity of 1200 mU/mg with (*S*)-1-PheOH under standard assay conditions (while with 10 mM benzyl alcohol, 1 mM NADP^+^, 50 mM glycine-KOH, pH 10.0, the activity was 2300 mU/mg). The aspecific van der Waals interactions provided by the two Phe residues could explain the promiscuous activity of *Hv*ADH2 on aromatic substrates.

Although flurbiprofenol has significantly increased bulk in the side chain, the docking analysis shows that the mode of binding is very similar to (*S*)-1-PheOH. This is because the additional aryl ring of the ligand is placed at the active site entrance, in contact with bulk solvent. The hydroxyl group of the substrate is still within H-bonding distance to S40 and is 3.7 Å from the active site Zn^2+^ ion. The activity of purified wild-type *Hv*ADH2 toward 1 mM *rac*-flurbiprofenol (2 M KCl, 30% MeOH to facilitate solubility) is 11.1 mU/mg. The racemic mixture of this alcohol was used since it was produced by the synthetic procedure employed (S1 File). Based on the *Hv*ADH2-substrate complex model, this >100-fold drop in specific activity relative to (*S*)-1-PheOH could be explained by non-optimal positions of the reactive carbon atom of the substrate and the C4 of the cofactor NAD^+^ (Fig 1C). Mutagenesis of F85 and F108 (i.e., substitution with a smaller residue) could result in a shorter distance between these two reactive atoms, thus affecting the catalytic activity of the enzyme by facilitating hydride transfer.

#### Sequence alignment analysis

**Fig 2:**
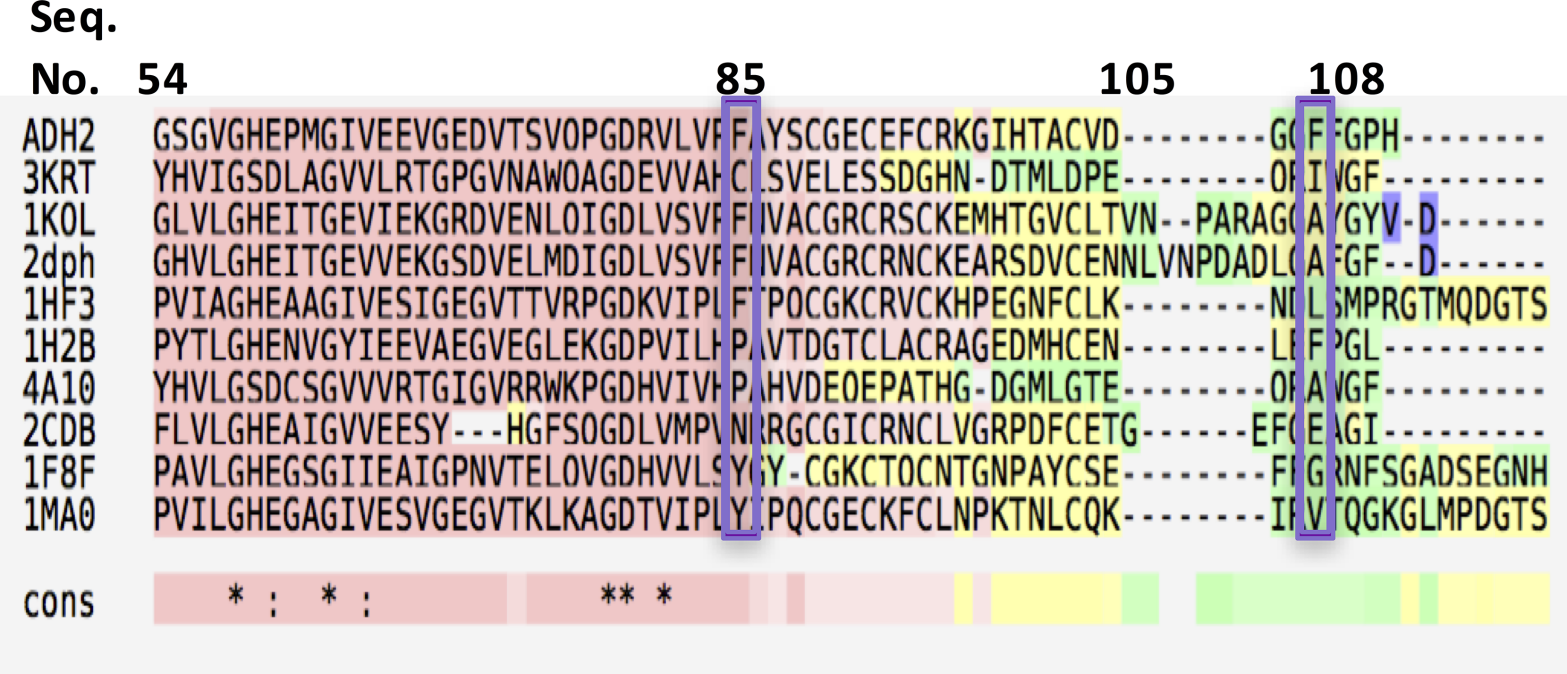
Sequence alignment of HvADH2 with top 9 homologues. *3KRT*: *putative crotonyl CoA reductase from Streptomyces coelicolor*. *1KOL*: *Formaldehyde dehydrogenase from Pseudomonas putida*. *2DPH*: *Formaldehyde dismutase from Pseudomonas putida*. *1HF3*: liver alcohol dehydrogenase from *Equus caballus*. *1H2B alcohol dehydrogenase from Aeropyrum pernix*. *4A10*: *2-octenoyl-CoA carboxylase reductase from Streptomyces sp*. *JS360*. *2CDB*: *glucose dehydrogenase from Sulfolobus solfataricus*. *1F8F*: *benzyl alcohol dehydrogenase from Acinetobacter calcoaceticus*. *1MAO*: *glutathione-dependent formaldehyde dehydrogenase from Homo sapiens*.

Bio-prodict 3DM database was used for the alignment of over 14000 sequences (*Hv*ADH2 no. D4GP73) to determine the conservation degree of the two identified phenylalanine residues (top 9 homologues shown in Fig 2) [25]. Across the alignment, the amino acid distribution at position 88 (numbering is per the 3DM database corresponding to F85 in *Hv*ADH2) was 17% phenylalanine, 16% proline and 28% was a gap. Position i1e (corresponding to F108) was less conserved in the alignment since it was found in only a limited pool of 400 sequences: the percentage of phenylalanine residues at this position was 9% (39% lysine, 21% arginine, and 9% phenylalanine with no gaps). Both positions seem to show a low degree of conservation pointing to a role in definition of the enzyme’s substrate preference: both positions were subjected to mutagenesis to investigate their role in substrate binding.

### Site-directed mutagenesis of F85 and simultaneous site saturation of F85 and F108

To investigate the significance of F85, four mutations were investigated: a conservative F85Y variant as well as more challenging F85A, F85V and F85R substitutions. F85Y was the only variant which retained activity and was tested with a range of substrates, including flurbiprofenol. Activities were determined for *Hv*ADH2 wild-type and F85Y variant crude lysates; substrate concentration was fixed at 10 mM for benzyl alcohol and enantiopure molecules or 20 mM for racemic ones. No activity was detected with flurbiprofenol and the variant enzyme. The reference 100% activity with BzOH and wild-type *Hv*ADH2 refers to 247 mU/mg whereas 280 mU/mg with F85Y. In all cases, activity for F85Y variant was lower compared to wild-type: F85Y retained 50% activity with (*S*)-1-PheOH, whereas wild-type retained 73%. F-MBA was poorly accepted by both wild-type (17%) and F85Y (9%). While 2-Phe-1-Prop was an excellent substrate for wild-type (98%), activity dropped with F85Y (16%). Wild-type activity with 4-Phe-2-But was 53% compared to just 8% with F85Y.

Site-saturation mutagenesis was also performed at both sites, F85 and F108, simultaneously and screened for activity with flurbiprofenol (S1 File). From multiple rounds of screening, a double variant identified as F85AF108G was isolated as the best hit, and the substrate scope was further investigated. Docking (*S*)-flurbiprofenol with F85AF108G showed the correct conformation of the distal phenyl ring. Activity of HvADH2 F85AF108G in the crude extract was 30% higher with flurbiprofenol when compared to wild-type but when purified, this activity diminished and was therefore deemed a false positive. Since site directed and site saturation of F85 did not yield an improved variant, we focused on F108.

### Site directed mutagenesis of F108 and screening with flurbiprofenol

Mutations at position F108 were evaluated initially by *in silico* modelling and docking with (*S*)*-*flurbiprofenol. *Hv*ADH2 variants F108A, F108G, F108L, F108P, F108V, F108W, and F108Y were modelled and tested *in silico* for affinity with (*S*)-flurbiprofenol, the main differences in theoretical binding energy are reported in Table ES.2. F108V was predicted as the best variant because of its ability to accommodate the biphenyl moiety of (*S*)-flurbiprofenol (S9A and S9B Fig). *In silico* results obtained with F108W, F108Y, F108P, and F108L variants were also indicative of improved binding. F108A substitution (S9C and S9D Fig) appeared to open the binding pocket and allow the distal aryl ring to point towards bulk solvent, which was not optimal in the wild-type enzyme.

**Fig 3:**
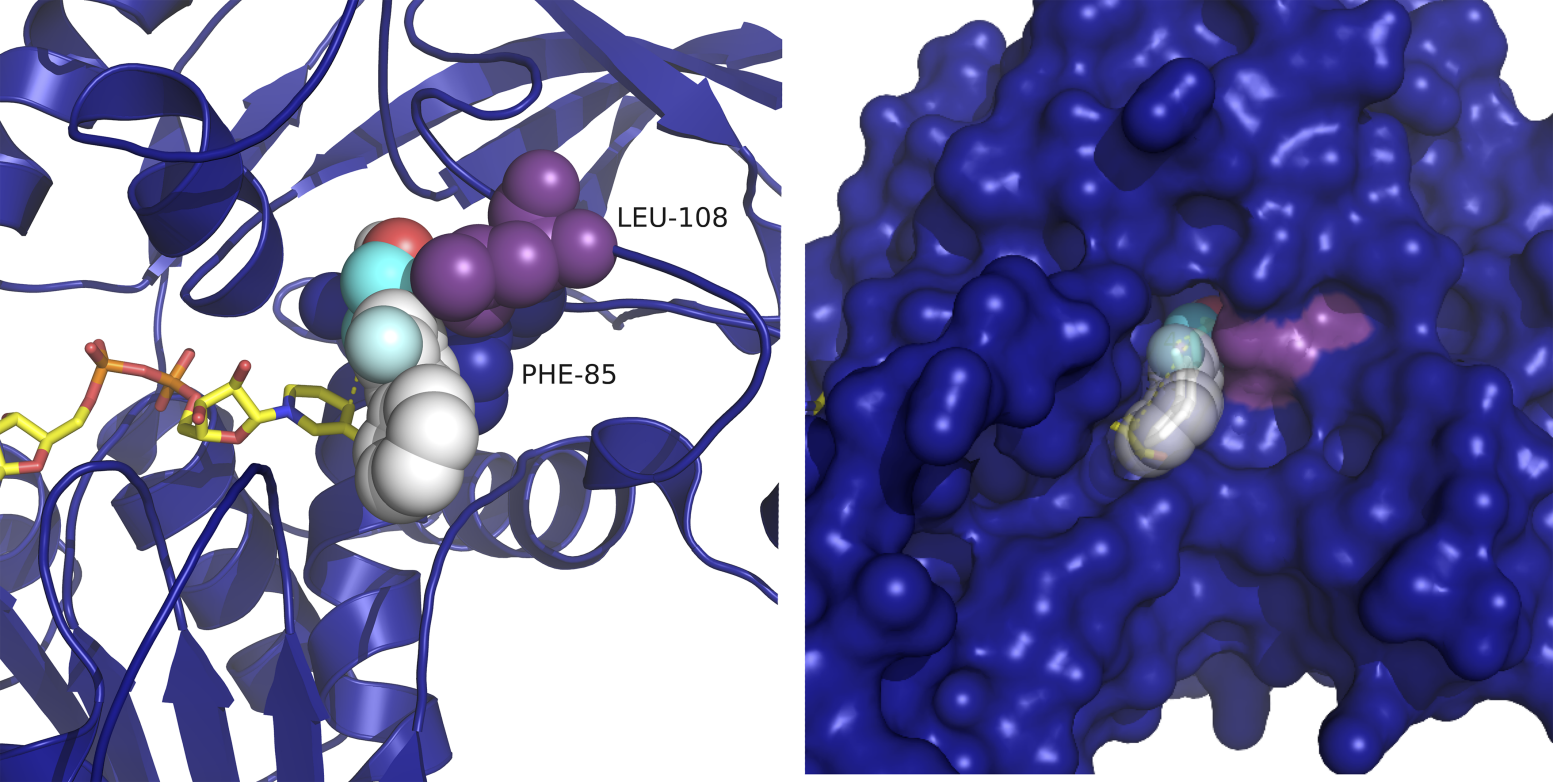
F108L docked (F85 blue spheres and L108 purple spheres; NAD^+^, yellow sticks) with (S)-flurbiprofenol (white spheres), surface view. The distance from the hydroxyl oxygen to the catalytic zinc (O-Zn) is 4.4 Å, and the distance from the substrate α-carbon to the C4 of the nicotinamide ring (αC-C4), is 5.9.

All these variants were engineered by site directed mutagenesis, expressed and purified (S1 File, small scale expression and purification) and activities are reported in Table 1. Contrary to the indications gathered from the docking, F108A and F108G showed no activity with *rac*-flurbiprofenol, whereas F108W, which introduces more steric bulk into the active site, was slightly more active than WT (13.8 vs. 11.1 mU/mg). The specific activity of F108Y variant (the most conservative substitution) on the latter compound was virtually identical to WT *Hv*ADH2. The F108L variant (docking shown in Fig 3) showed the highest specific activity of 25.4 mU/mg, a 2.3-fold increase in activity compared to WT enzyme. To investigate if a non-conserved mutation would be beneficial, a methionine variant was prepared (F108M). While F108M HvAFH2 was active with EtOH (1103 mU/mg), it did not show activity towards flurbiprofenol.

**Table 1:** Activity of purified wild-type and F108 variants of *Hv*ADH2 with *rac*-flurbiprofenol.

Variant	Specific activity (mU/mg protein)
**WT**	11.1 ± 0.2
**F108G**	0
**F108A**	0
**F108V**	16.2 ± 0.1
**F108W**	13.8 ± 0.3
**F108L**	**25.4 ± 0.2**
**F108Y**	11.7 ± 0.3
**F108P**	0
**F108M**	b.d.

b.d. = below detection

**Fig 4:**
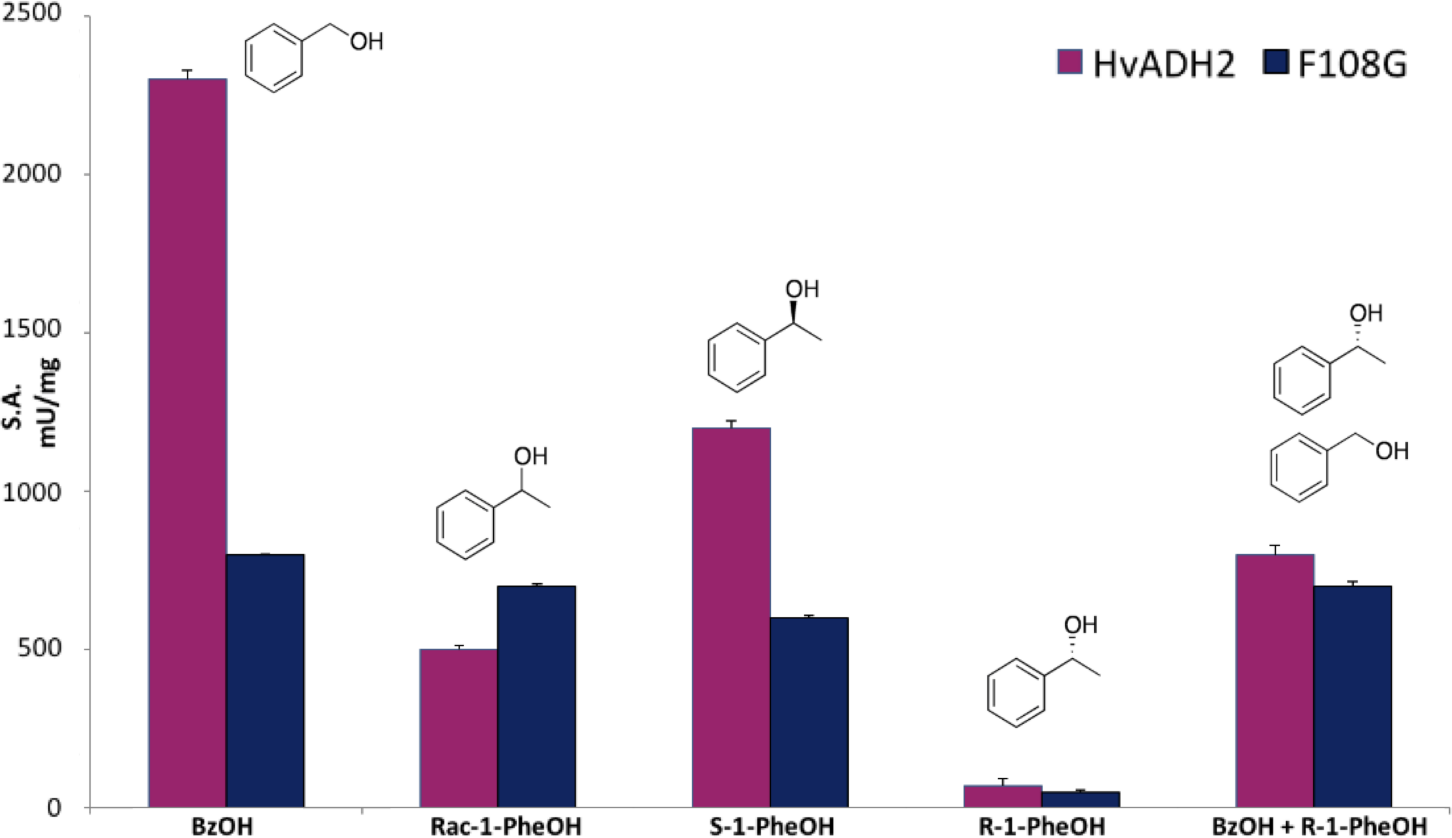
Substrate specificity of purified F108G variant compared to WT HvADH2. Buffer conditions: 4 M KCl, 50 mM Gly-KOH, pH 10.0.

In order to clarify the structure-function relationships modulating *Hv*ADH2, purified F108G variant and wild-type *Hv*ADH2 were assayed with BzOH, *rac*-1-PheOH, (*S*)-1-PheOH and (*R*)-1-PheOH (Fig 4). Even if F108G *Hv*ADH2 was not active with flurbiprofenol, the dramatic change induced in the active site yielded a fully folded protein with 40% activity with respect to the WT, i.e. 800 mU/mg with 10 mM benzyl alcohol (see S1 File for details about the expression and purification).

**Fig 5:**
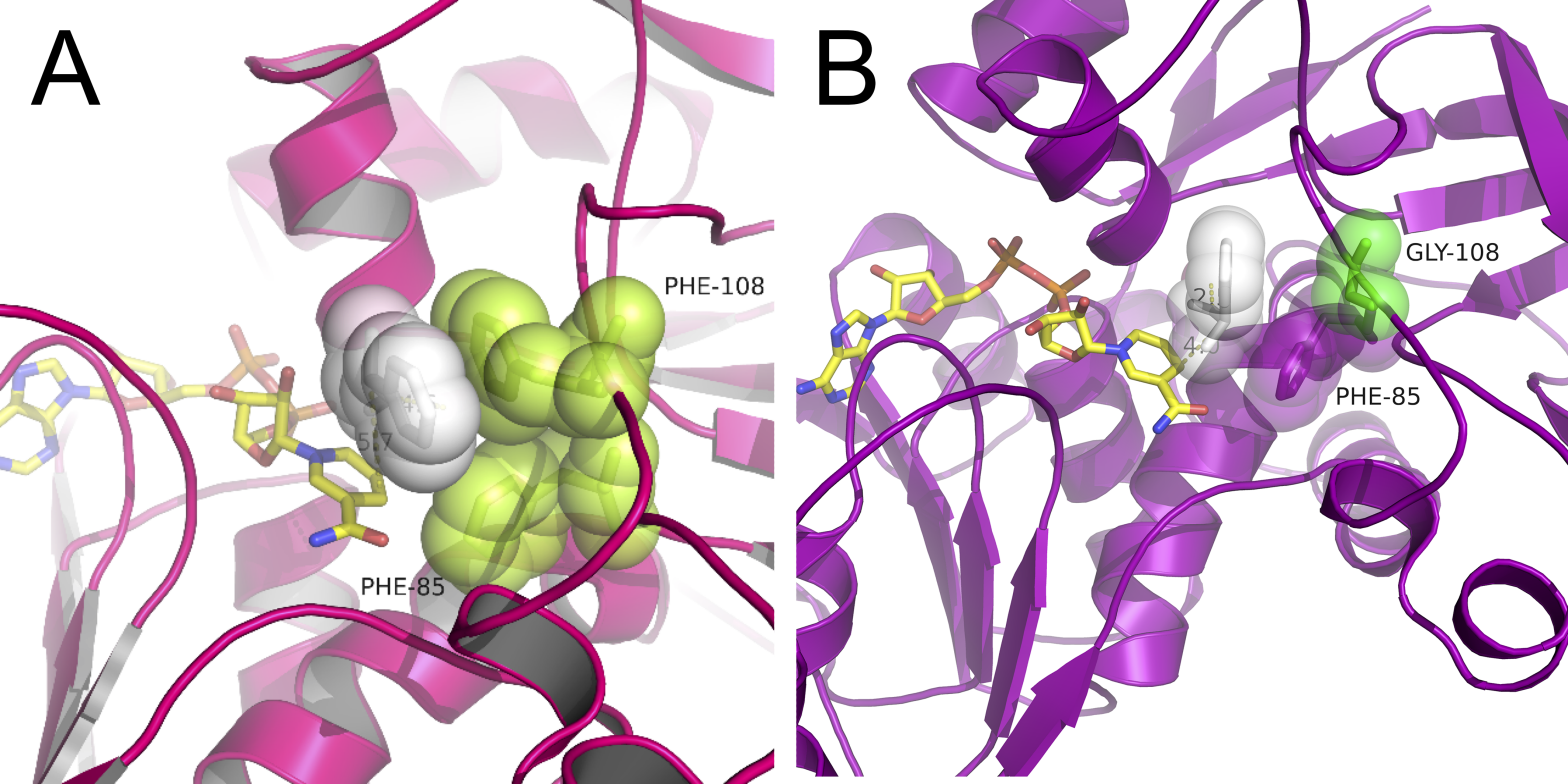
Docking of (R)-1-PheOH (white spheres). A) Catalytic site of the WT enzyme (F85 and F108 green spheres, NAD^+^, yellow sticks) shows stabilization of the substrate aromatic ring by F108. The distance from the hydroxyl oxygen to the catalytic zinc (O-Zn) is 4.6 Å, and the distance from the substrate α-carbon to the C4 of the nicotinamide ring (αC-C4), is 5.7 Å. B) Catalytic site of the F108G variant. The distance from the hydroxyl oxygen to the catalytic zinc (O-Zn) is 2.5 Å, and the distance from the substrate α-carbon to the C4 of the nicotinamide ring (αC-C4), is 4.0 Å.

Remarkably, F108G showed an increase in activity with *rac*-1-PheOH with respect to the WT (680 and 460 mU/mg respectively). However, with optically pure (*S*)-1-PheOH, WT was clearly more active (1200 mU/mg), whereas F108G maintained almost unaltered activity (620 mU/mg). To investigate if the opposite enantiomer was accepted as substrate, (*R*)-1-PheOH was tested with both WT and F108G *Hv*ADH2 but showed negligible activity (70 and 50 mU/mg, respectively). Addition of 10 mM of (*R*)-1-PheOH to the standard reaction mixture (10 mM benzyl alcohol) reduced the WT *Hv*ADH2 specific activity down to 840 mU/mg (in comparison to the 2,300 mU/mg of the original one), while, in the case of the F108G variant, the activity was virtually unaffected. Docking of (*R*)-1-PheOH to wild-type *Hv*ADH2 shows a clear interaction between the aromatic side chain of the substrate and F108 (Fig 5A) which is not present in the variant harbouring F108G (Fig 5B). On closer inspection of the docking, in the wild-type model, the distance between the reactive carbon atom of the substrate and the C4 of the cofactor NAD^+^ is 5.7 Å. This distance is shortened to 4.2 Å in the F108G model. The experimental evidence together with the *in silico* predictions strongly suggest that removal of the bulky side-chain from F108 in the glycine variant creates a cavity in the active site. This space could allow the binding of the preferred enantiomer while still housing the (*R*)-1-PheOH without hampering catalytic efficiency of the enzyme in the presence of a racemic mixture.

## Experimental

### Expression and purification of wild-type and variants of *Hv*ADH2 in *Haloferax volcanii*

The transformation, production, purification and identification of wild-type and variants of *Hv*ADH2 were performed as described previously [16, 17]. Small scale expression and purification are detailed in S1 File.

### Enzyme assays

Enzyme activity was assayed as production of the NADPH cofactor detected at 340 nm, measured in intervals of 1 min for 20 min at 50°C (Epoch 2 microplate reader, BioTek, Bad Friedrichshall, Germany; 96 Well Clear Flat Bottom UV-Transparent Microplate Corning®, 3635). All kinetic assays were performed in triplicate. The blank was treated by adding the storage buffer (3 M KCl, 100 mM Tris-HCl, pH 8.0) instead of enzyme.

### *Hv*ADH2 homology modelling

The web-based server SWISS-MODEL was used to build the model of *Hv*ADH2 with a GMQE score of 0.68 [20, 21]. The details for the docking and inspection are mentioned in S1 File.

### *Hv*ADH2 mutant generation

The *adh2* gene harboured in the pTA963 plasmid was mutated using the QuikChange Lightning Multi Site-Directed Mutagenesis Kit provided by Agilent Technologies®. Details of the PCR reaction and the oligonucleotide primers are detailed in S1 File and S1 Table.

## Conclusions

Rational design coupled with molecular modelling were applied here for the generation of several site directed variants of *Hv*ADH2. Building a homology model of *Hv*ADH2 allowed the identification of two phenylalanine residues, at position 85 and 108, which were proposed to be critical residues for the binding of 1-PheOH due to π-π stacking interactions. Docking (*S*)-flurbiprofenol (an intermediate for the non-steroidal anti-inflammatory drug, flurbiprofen) into the wild-type *Hv*ADH2 model showed the unfavourable conformation of the distal aryl ring due to the interactions with the two Phe residues.

F85 appears critical for the stabilization in the binding pocket of small aromatic substrates, whereas the absence of the side chain of F108 facilitates the binding of secondary aromatic alcohols (i.e., 1-PheOH). Site saturation mutagenesis was performed at both sites to make a small, diverse library. However, several rounds of screening failed to identify an improved variant. The best hit, F85AF108G lost all activity after purification. It was then decided to perform site directed mutagenesis at each site, independently of each other. F85 tolerated only conservative mutations such as F85Y which was active with all tested substrates albeit less than the WT *Hv*ADH2. Sequence alignments confirmed that F108 was indeed a less conserved position and site directed mutagenesis was performed following *in silico* modelling and docking to predict improved variants. Among the generated single point variants, F108W, F108Y and F108L accepted flurbiprofenol with enhanced activity compared to wild-type *Hv*ADH2; specifically, F108L had a 2.3-fold improvement in activity. The F108G variant showed surprisingly no activity with flurbiprofenol while retaining 40% of the WT activity with benzyl alcohol. Further testing of this variant with *rac*-1-PheOH indicated that the enzyme performed significantly better than the WT possibly due to the larger binding site created. (*R*)-1-PheOH is not a substrate for the glycine variant nor for the WT *Hv*ADH2. This compound is a strong competitive inhibitor of the latter; whereas it is not able to bind to the F108G variant possibly due to the key role of F108 in stabilising the aromatic moiety of the substrate. *In silico* docking and site directed mutagenesis were successfully applied to improve enzymatic activity with a bulky, aromatic substrate which was poorly accepted by the wild-type enzyme. This model-guided mutagenesis was performed on a protein for which the closest structural analogue had less than 30% similarity in the sequence, underlying the power of this technique.

## Supporting information

S1 File (PDF)

S1 Table Mutant primer sequences. (TIFF)

S2 Table *(S)-*flurbiprofenol docking energies (kcal/mol) determined by the simulation software Autodock Vina. (TIFF)

S1 Scheme Esterification of flurbiprofen and reduction to the corresponding alcohol. (TIFF)

S1 Fig Quality of the HvADH2 model. Regions of the structure that can be rejected at the 95% and 99% confidence level are represented in yellow and red respectively.
(TIFF)

S2 Fig Verified 3D plot of HvADH2 model. (TIFF)

S3 Fig ^1^H NMR spectrum of ethyl 2-(2-fluoro-[1,1'-biphenyl]-4-yl)propanoate. (TIFF)

S4 Fig ^13^C NMR spectrum of ethyl 2-(2-fluoro-[1,1'-biphenyl]-4-yl)propanoate. (TIFF)

S5 Fig ^1^H NMR spectrum of 2-(2-fluoro-biphenyl-4-yl)-propan-1-ol. (TIFF)

S6 Fig ^13^C NMR spectrum of 2-(2-fluoro-biphenyl-4-yl)-propan-1-ol. (TIFF)

S7 Fig HvADH2 substrate specificity with aromatic ketones at pH 8.0 and 10.0. Substrate concentration was fixed at 10 mM in 4 M KCl, 50 mM glycine buffer, pH 10.0.
(TIFF)

S8 Fig SDS-PAGE gel of HvADH2 variants purification on a Ni-NTA mini-column. Lane 1: broad range protein marker Precision Plus Kaleidoscope, (10–250 kDa); Lane 2: WT; Lane 3: F108Y; Lane 4; F108L Lane 5; F108W.
(TIFF)

S9 Fig **A-D. Docking analysis of *(S)-*flurbiprofenol to F108x HvADH2 variants**. Panel A: Docking of (S)-flurbiprofenol to F108V HvADH2; panel B: surface view of panel A. The distance from the hydroxyl oxygen to the catalytic zinc (O-Zn) is 4.3 Å, and the distance from the substrate α-carbon to the C4 of the nicotinamide ring (αC-C4), is 6.7; panel C: docking of (S)-flurbiprofenol to F108A HvADH2; panel D: surface view of panel C. F85 is represented in purple spheres and F108 by lilac spheres, NAD^+^ by yellow sticks and (S)-flurbiprofenol by white spheres. The distance from the hydroxyl oxygen to the catalytic zinc (O-Zn) is 4.8 Å, and the distance from the substrate α-carbon to the C4 of the nicotinamide ring (αC-C4), is 7.1.
(TIFF)

S10 Fig SDS-PAGE analysis of HvADH2 F108G purification from *Haloferax volcanii* strain H1325. Lane 1: broad range protein marker P7702S, (2–212 kDa); Lane 2: crude lysate; Lane 3–10: eluted fractions 1–8 respectively. The band corresponding to F108G is indicated by the arrow.
(TIFF)

## Abbreviations

ADH: alcohol dehydrogenase
F-MBA: fluoro-methyl benzyl alcohol
2-Phe-1-prop: 2-phenyl-1-propanol
4-Phe-2-But: 4-phenyl-2-butanol
WT: wild-type
*Hv*ADH2: ADH2 from *Haloferax volcanii*
