# Supplementary material for: Engineering substrate promiscuity in halophilic alcohol dehydrogenase (HvADH2) by in silico design
Source: PLoS One. 2017 Nov 30;12(11):e0187482. doi: 10.1371/journal.pone.0187482 (PMC5708825; doi:10.1371/journal.pone.0187482)
Supplement: S1 File — (PDF) [file pone.0187482.s001.pdf]

Supporting Information

Engineering substrate promiscuity in halophilic alcohol dehydrogenase (*Hv*ADH2*)* by *in silico* design

Jennifer Cassidy[1], Larah Bruen[1], Elena Rosini[2,3], Gianluca Molla[2,3], Loredano Pollegioni[2,3] and Francesca Paradisi[1,4*]

[1] *Synthesis and Solid State Pharmaceutical Centre (SSPC), School of Chemistry, University College Dublin, Belfield, Dublin 4, Ireland*

[2] *Dipartimento di Biotecnologie e Scienze della Vita, Università degli Studi dell'Insubria, Varese, Italy*

[3] *The Protein Factory, Politecnico di Milano, Università degli Studi dell'Insubria, Milano, Italy*

[4] *School of Chemistry, University Park, University of Nottingham, Nottingham, NG7 2RD, UK*

***Corresponding author**

Francesca Paradisi

E-mail: Francesca.Paradisi@nottingham.ac.uk

Telephone: 0115 74 86267

Address: School of Chemistry, University Park, University of Nottingham, Nottingham NG7 2RD, UK

Table of contents

1 – Materials

2 – Mutant primers

3 – Mutant PCR conditions

4 – Model quality and docking of *Hv*ADH2

5 – Synthesis of flurbiprofenol

6 – *Hv*ADH2 substrate specificity towards aromatic ketones

7 – Small scale expression and purification of WT and mutants

8 – Docking of F108V and F108A

9 – F108G characterisation

1 - Materials

The cofactors, $NADP^+$ and NADPH were purchased from Apollo Scientific Ltd, UK. (*S*)-1-Phenylethanol was purchased from Alfa Aesar, (*R*)-1-phenylethanol was purchased from Acros. Racemic 1-phenylethanol, 2-phenyl-1-propanol, 4-phenyl-2-butanol were purchased from Santa Cruz Biotechnology. Diisobutylaluminum hydride and sodium borohydride were purchased from Sigma Aldrich.

2 - Mutagenic primers

Mutant primers were designed exploiting the QuikChange Primer Design tool (Agilent Technologies®) and were synthesized from Eurofins Genomics® as shown is S1 Table.

3 - Mutagenic PCR conditions and INT/PES screening

The PCR mixture contained 10 x QC Multi Reaction Buffer (2.5 µL), Quik Solution (0.75 µL), template DNA pTA1205 (2 µL, 100 ng), adh2F85mut primer (1 µL, 100 ng/µL), or adh2F108satmut primer (1 µL, 100 ng/µL), dNTP mix (1 µL), QC Multi Enzyme Blend (1 µL) and ddH_2O (15.75 µL). It employed an initial denaturation temperature of 95 °C for 2 minutes. This was followed by 30 cycles of denaturation at 95 °C for 20 seconds, primer annealing at 57 °C (F85Y, F85A, F85R, F85V, F108L, F108A, F108G, F108Y, F108W, F85F108 saturation) or 65 °C (F108V), 68 °C (F108P) or 72 °C (F108M) for 30 seconds and primer extension at 65 °C for 30 seconds/kb of plasmid length (in this case, 4 min 50 s). A final extension at 65 °C was applied for 5 minutes. To ensure that only mutated DNA was present, DpnI restriction enzyme was added to each amplification reaction and the reaction was incubated at 37 °C for 50 minutes. A modified version of the INT/PES screen for dehydrogenase was applied to the saturation library [1]. This involves the production of the red insoluble formazan. 2-(4-iodophenyl)-3-(4-nitrophenyl)-5-phenyltetrazolium chloride hydrate (INT) in the presence of phenazine ethosulfate (PES) reacts with NAD(P)H produced by alcohol dehydrogenase to form the insoluble formazan. This screening method was applied in 96 well plates to detect a variant of *Hv*ADH2, which catalyzed the oxidation of (*R,S*)-flurbiprofenol. Controls, pTA963 (empty plasmid) and wild-type were added into two wells of each screening plate. Reagents were mixed together at RT and transferred to 96 well plates. The plates were incubated in the dark before the addition of supernatant, after a short exposure to light, plates were incubated at 37 °C for 1 hour. A strong red colour was indicative of a hit, which could then be recovered from the replica plate.

4 – Model quality and docking of *Hv*ADH2

MacPyMol was used for structural evaluation and docking inspection (open source licence). AutoDock Tools was used to prepare the protein and the ligand (substrates of interest). All water molecules and ligands except the NAD^+ cofactor and the Zn ion. were removed from the structure. the charge of the catalytic Zn-ion was set to +2). AutoDock Vina executed the docking experiments (open source licence, http://vina.scripps.edu/) [2]. During the docking procedure, the position, orientation and torsion angles of the ligand were allowed to vary, while the protein was kept rigid. The grid for docking was centered on the NAD C4N,

coordinates X, -2.9; Y, 1.6; and Z, -0.61 and exhaustiveness of 64. PDBeChem was used to download substrate structures. Substrates, which were unavailable from PDBeChem, were designed using Avogadro software. The models of the structures of the *Hv*ADH2 variants were obtained using the "Mutagenesis Wizard" of PyMol. Maestro version 10.4.017 (Schrodinger LLC, New York, NY, USA) was used to minimize the local energy of the variant models in the region of the introduced mutation.

The quality of the model was also checked using the software Verify 3D [3]. A reliable model should have at least 80% of its amino acids higher than the threshold limit (≥ 0.2). In the case of *Hv*ADH2 model, almost all residues of the model (96.6%) had an averaged 3D-1D score ≥ 0.2 (S1-2 Figs.). Docking energies of (*S*)-flurbiprofenol with each variant were evaluated with Autodock Vina (S2 Table).

5 - Synthesis of flurbiprofenol

The target substrate for screening is the alcohol precursor of Flurbiprofen. A recent report describes an efficient oxidative kinetic resolution from the racemic Profen aldehyde to the carboxylic acid [4]. Flurbiprofen was synthesized in 100% yield with two equivalents of acetone, from the starting aldehyde using acetone as a sacrificial cosubstrate. An important note from this study is that the synthesis of flurbiprofen from the alcohol precursor was not determined and therefore is of potential interest. The alcohol precursor of flurbiprofen (flurbiprofenol) was synthesized following standard procedures in two steps by esterification of the carboxylic acid followed by reduction with $LiAlH_4/Et_2O$. Racemic flurbiprofenol was prepared as described in S1 Scheme by modifying the method of Giacomini *et al* [5].

Starting from the corresponding commercial carboxylic acids, preliminary esterification was carried out with BF_3/Et_2O in ethanol and then $LiAlH_4$ reduction in Et_2O to the alcohol. Racemic 2-fluoro-α-methyl-4-biphenylacetic acid, Flurbiprofen, (1.23 mmol, 300 mg) was refluxed in EtOH (5 mL) in the presence of 1 equivalent of $BF_3.Et_2O$ (0.1 mL). At disappearance of the starting carboxylic acid, the solvent was concentrated, the crude dissolved in EtOAc (10 mL) and washed with aqueous $NaHCO_3$, the organic phase was

concentrated furnishing ethyl 2-(2-fluoro-[1,1'-biphenyl]-4-yl)-propanoate in 83% yield. Ethyl 2-(2-fluoro-[1,1'-biphenyl]-4-yl)propanoate ^{1}H NMR (500 MHz, $CDCl_3$): δ 1.24 (t, *J* = 7.1 Hz, 3H), 1.53 (d, *J* = 7.2 Hz, 3H), 3.73 (q, *J* = 7.2 Hz, 1H), 4.16 (qq, *J* = 10.9, 7.1 Hz, 2H), 7.10 – 7.14 (m, 2H), 7.32 – 7.40 (m, 4H), 7.50 – 7.53 (m, 2H), S3 Fig. Ethyl 2-(2-fluoro-[1,1'-biphenyl]-4-yl)propanoate ^{13}C NMR (126 MHz, $CDCl_3$) δ 14.13, 18.44, 45.07, 60.96, 115.12, 115.31, 123.48, 123.51, 127.62, 127.69, 127.79, 128.41, 128.92, 128.94, 130.72, 130.75, 135.54, 141.95, 142.02, 158.69, 160.66, 173.96, S4 Fig.

$LiAlH_4$ (1.4 mmol, 54 mg) was weighed into a two neck round bottom flask under inert atmosphere. The ethyl ester (0.92 mmol, 250 mg), dissolved in dry Et_2O (4 mL) then added dropwise. After 1 h reflux reaction was complete, as monitored by TLC. Reaction was cooled to 0 °C and a saturated solution of Na_2SO_4 was added dropwise until fizzing stopped. The suspension was filtered under suction over Celite and rinsed with Et_2O. The organic phase was dried and concentrated under vacuum and 2-(2-fluoro-[1,1'-biphenyl]-4-yl)propan-1-ol was obtained after purification by flash chromatography (1:1 EtOAc:Toluene) in 65 % yield. 2-(2-fluoro-biphenyl-4-yl)-propan-1-ol (Flurbiprofenol) ^{1}H NMR (500 MHz, $CDCl_3$): δ 1.30 (d, *J* = 7.0, 3H, CH_3), 3.00 (sextet, *J* = 7.0, 1H, CH), 3.74 (d, *J* = 7.0, 2H, CH_2), 7.05–7.16 (m, 2H, arom), 7.28–7.61 (m, 6H, arom), S5 Fig.; 2-(2-fluoro-biphenyl-4-yl)-propan-1-ol (Flurbiprofenol) ^{13}C NMR (126 MHz, $CDCl_3$) δ 17.47, 42.01 (d, *J* = 1.4 Hz), 68.43, 114.97 (d, *J* = 23.0 Hz), 123.49 (d, *J* = 3.4 Hz), 127.02, 127.53, 127.88, 128.41, 128.92 (d, *J* = 2.9 Hz), 130.76 (d, *J* = 4.1 Hz), 135.67, 145.52 (d, *J* = 7.0 Hz), 160.83, S6 Fig.

6 – *Hv*ADH2 substrate specificity towards aromatic ketones

*Hv*ADH2 accepted a range of primary and secondary aromatic alcohols. The substrate specificity of *Hv*ADH2, in the reductive direction is shown in S7 Fig. Importantly, *Hv*ADH2 accepted 2-phenylpropionaldehyde, gaining access to 2-arylpropanols.

7 – Small scale expression and purification of *Hv*ADH2 wild-type and variants

Small scale expression of *Hv*ADH2 variants was performed using *Haloferax volcanii* strain H1325 in 10 mL Hv-YPC media with shaking at 45 °C. After 24 h, cultures were spun down at 4,000 rpm for 10 min and resuspended in 9 mL Hv-YPC media and 1 mL of L-Trp (50 mM) and grown for a further 15 h. Cultures were harvested and resuspended in 750 µL of binding buffer (2 M NaCl, 20 mM imidazole, 20 mM Tris-HCl, pH 8.0). The crude suspension was sonicated with a microtip in 1 s pulses for 2 x 30 s and cell debris separated by centrifugation at 14,000 rpm, 4 °C for 30 min. The crude lysate was loaded onto a gravity Bio-Rad™ Micro Bio-spin column preloaded with 100 µL Thermo Scientific™ HisPur™ Ni-NTA resin. The resin was equilibrated in binding buffer and 6 mL of binding buffer was run through the column and elution buffer, containing 50 mM EDTA was run through in 3 x 100 µL steps. Elution three was the most pure and active fraction and was used for kinetic testing. Eluted fractions were assayed immediately and without dialysis. The SDS-PAGE analysis of the purified preparation is shown in S8 Fig. *Hv*ADH2 wild-type was also expressed in 10 mL culture and purified in the same manner for direct comparison.

8 – Docking of F108V and F108A

(*S*)-flurbiprofenol was docked into the *in silico* designed structural models of F108V and F108A variants as shown in S9 A-D Fig. respectively.

9 – F108G *Hv*ADH2 characterisation

F108G *Hv*ADH2 expression was scaled-up to 300 mL; the enzyme was purified by IMAC, as previously described [6]. F108G is therefore compared directly to purified WT *Hv*ADH2. The SDS-PAGE analysis of the fractions eluted during the enzyme purification is shown S10 Fig.

References

1. Chen S, Engel PC. Efficient screening for new amino acid dehydrogenase activity: Directed evolution of *Bacillus sphaericus*phenylalanine dehydrogenase towards activity with an unsaturated non-natural amino acid. J Biotechnol 2009; 142: 127-134.
2. Trott O, and Olson AJ. AutoDock Vina: improving the speed and accuracy of docking with a new scoring function, efficient optimization and multithreading. J Comput Chem. 2010; 31: 455-461.
3. Lüthy R, Bowie JU, Eisenberg D. Assessment of protein models with three-dimensional profiles. Nature. 1992; 356(6364): 83-5.
4. Könst P, Merkens H, Kara S, Kochius S, Vogel A, Zuhse R, Holtmann D, Arends IW, and Hollmann F. Enantioselective oxidation of aldehydes catalyzed by alcohol dehydrogenase. Angew Chem Int Ed Engl. 2012; 51: 9914-9917.
5. Galletti P, Emer E, Gucciardo G, Quintavalla A, Pori M, and Giacomini D. Chemoenzymatic synthesis of (2 S)-2-arylpropanols through a dynamic kinetic resolution of 2-arylpropanals with alcohol dehydrogenases. Org Biomol Chem. 2010; 8: 4117-4123.
6. Timpson LM, Alsafadi D, Mac Donnchadha C, Liddell S, Sharkey MA and Paradisi F. Characterization of alcohol dehydrogenase (ADH12) from *Haloarcula marismortui*, an extreme halophile from the Dead Sea. Extremophiles. 2012; 16: 57-66.
