# Supplementary figures and images for: Engineering substrate promiscuity in halophilic alcohol dehydrogenase (HvADH2) by in silico design
Source: PLoS One. 2017 Nov 30;12(11):e0187482. doi: 10.1371/journal.pone.0187482 (PMC5708825; doi:10.1371/journal.pone.0187482)

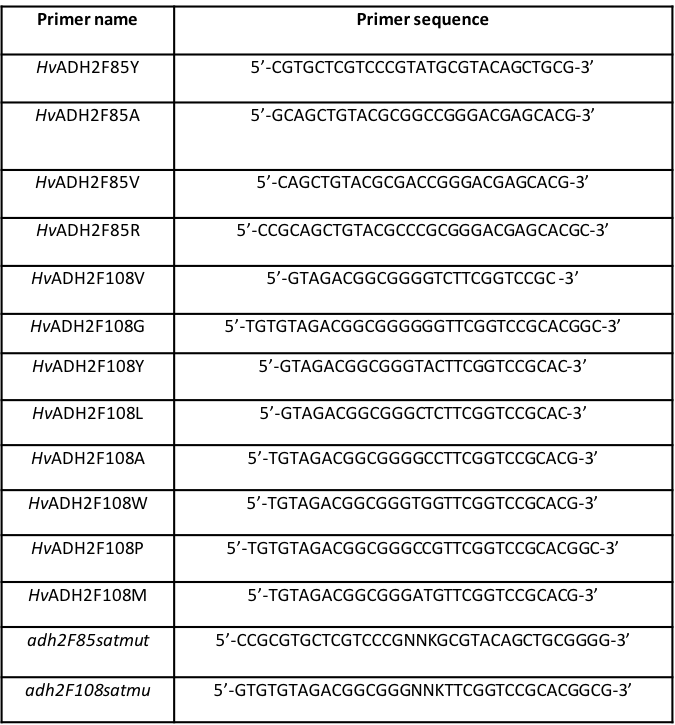

Supplement: S1 Table — (TIFF) [file pone.0187482.s002.tiff]

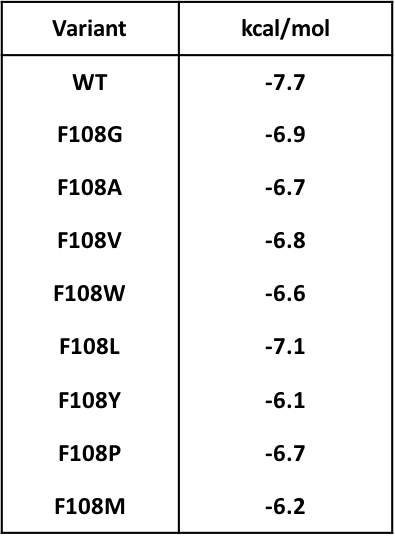

Supplement: S2 Table — (TIFF) [file pone.0187482.s003.tiff]

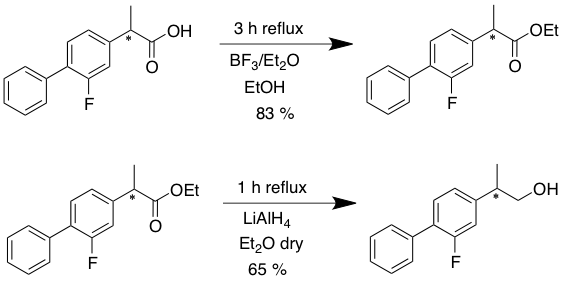

Supplement: S1 Scheme — (TIFF) [file pone.0187482.s004.tiff]

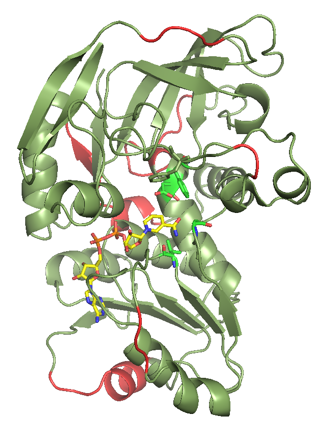

Supplement: S1 Fig — Regions of the structure that can be rejected at the 95% and 99% confidence level are represented in yellow and red respectively. (TIFF) [file pone.0187482.s005.tiff]

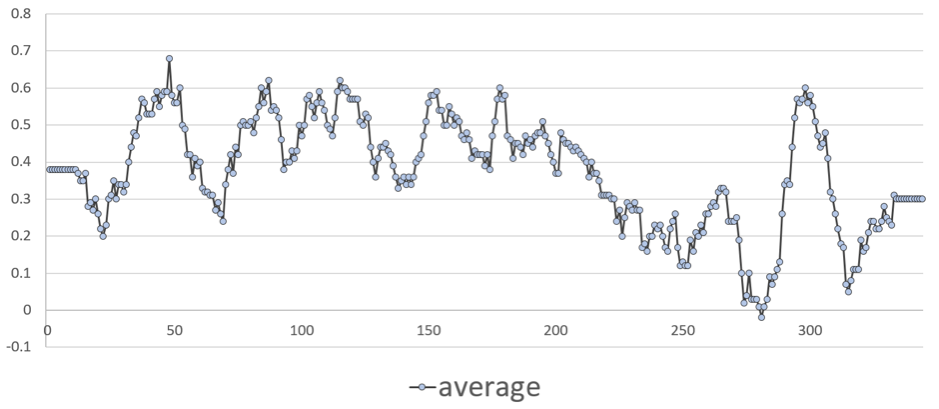

Supplement: S2 Fig — (TIFF) [file pone.0187482.s006.tiff]

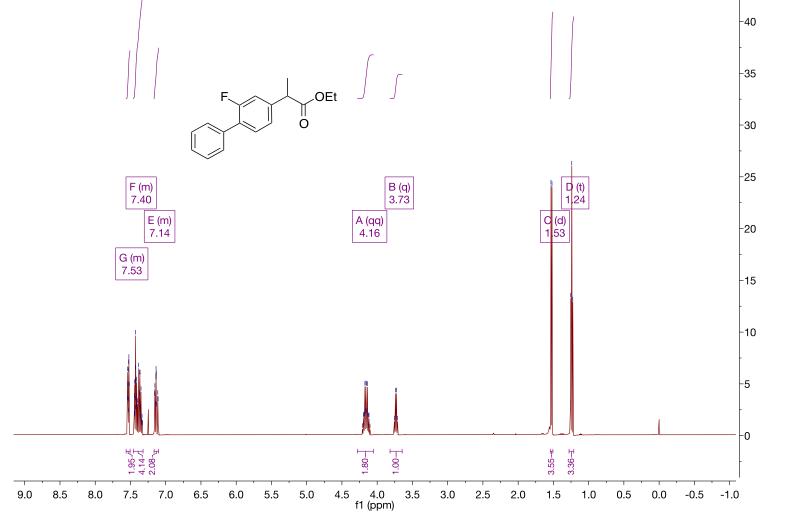

Supplement: S3 Fig — (TIFF) [file pone.0187482.s007.tiff]

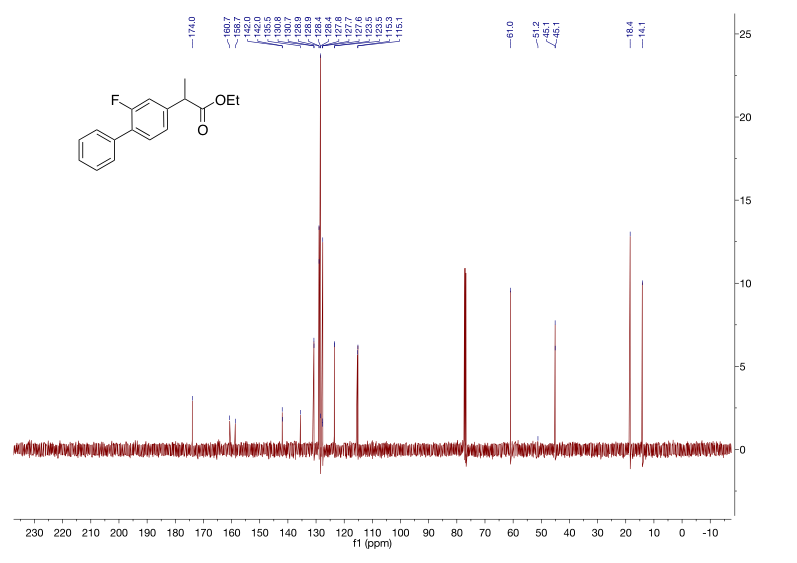

Supplement: S4 Fig — (TIFF) [file pone.0187482.s008.tiff]

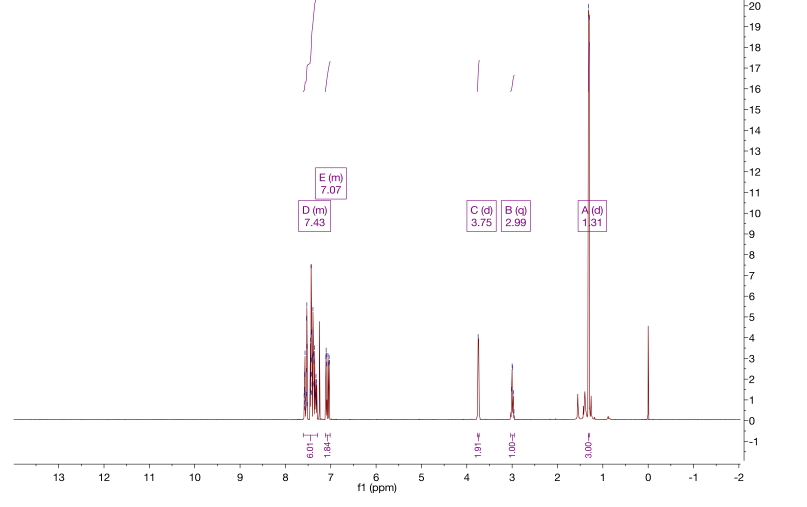

Supplement: S5 Fig — (TIFF) [file pone.0187482.s009.tiff]

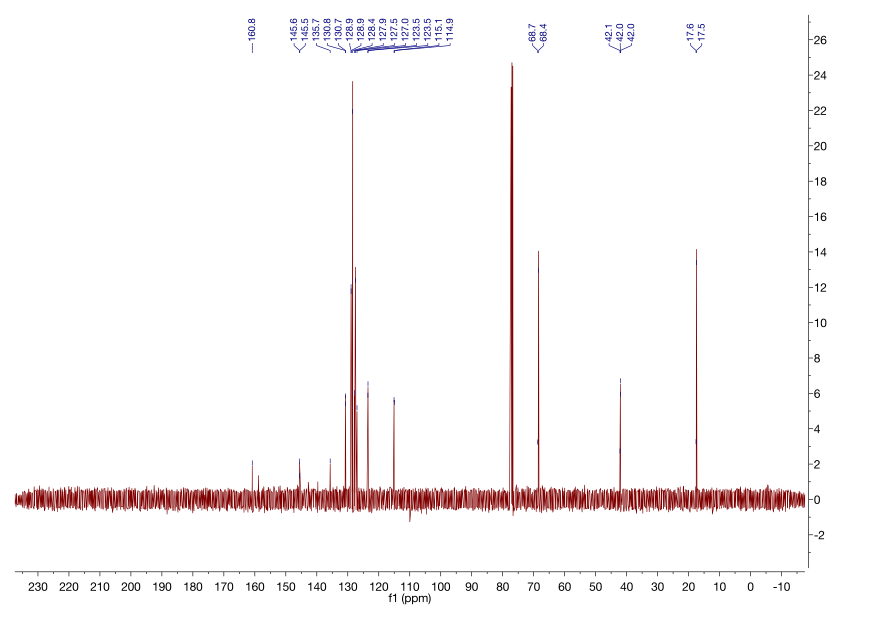

Supplement: S6 Fig — (TIFF) [file pone.0187482.s010.tiff]

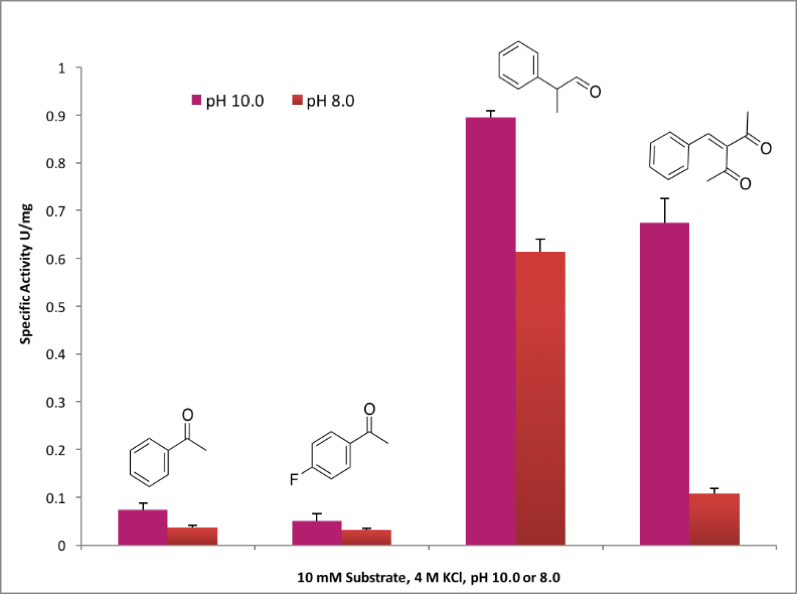

Supplement: S7 Fig — Substrate concentration was fixed at 10 mM in 4 M KCl, 50 mM glycine buffer, pH 10.0. (TIFF) [file pone.0187482.s011.tiff]

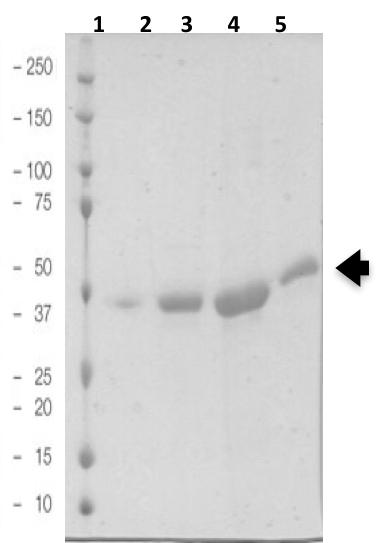

Supplement: S8 Fig — Lane 1: broad range protein marker Precision Plus Kaleidoscope, (10–250 kDa); Lane 2: WT; Lane 3: F108Y; Lane 4; F108L Lane 5; F108W. (TIFF) [file pone.0187482.s012.tiff]

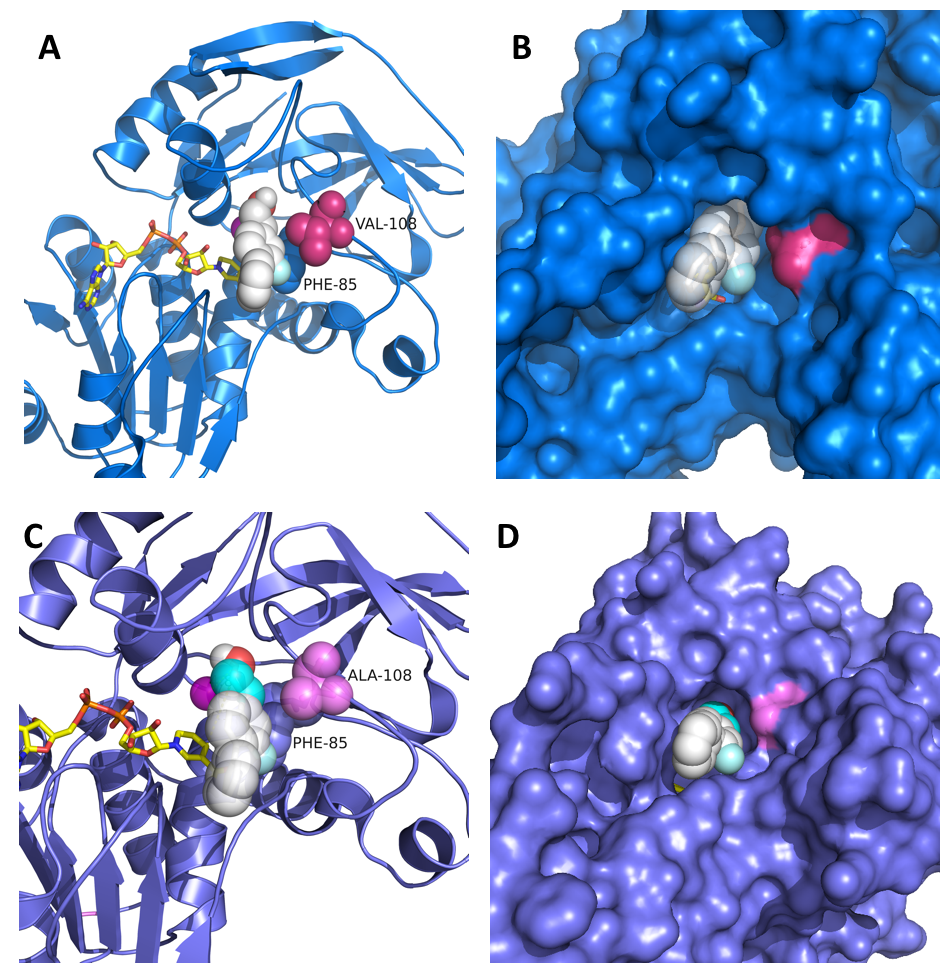

Supplement: S9 Fig — A-D. Docking analysis of (S)-flurbiprofenol to F108x HvADH2 variants. Panel A: Docking of (S)-flurbiprofenol to F108V HvADH2; panel B: surface view of panel A. The distance from the hydroxyl oxygen to the catalytic zinc (O-Zn) is 4.3 Å, and the distance from the substrate α-carbon to the C4 of the nicotinamide ring (αC-C4), is 6.7; panel C: docking of (S)-flurbiprofenol to F108A HvADH2; panel D: surface view of panel C. F85 is represented in purple spheres and F108 by lilac spheres, NAD+ by yellow sticks and (S)-flurbiprofenol by white spheres. The distance from the hydroxyl oxygen to the catalytic zinc (O-Zn) is 4.8 Å, and the distance from the substrate α-carbon to the C4 of the nicotinamide ring (αC-C4), is 7.1. (TIFF) [file pone.0187482.s013.tiff]

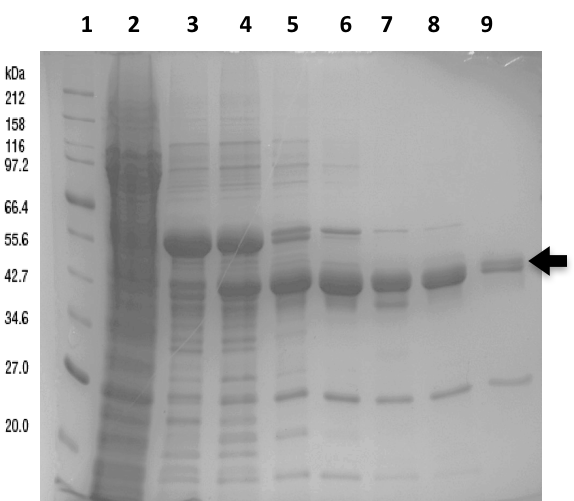

Supplement: S10 Fig — Lane 1: broad range protein marker P7702S, (2–212 kDa); Lane 2: crude lysate; Lane 3–10: eluted fractions 1–8 respectively. The band corresponding to F108G is indicated by the arrow. (TIFF) [file pone.0187482.s014.tiff]
